# Supplementary material for: An Exploratory Study on Seasonal Variation in the Gut Microbiota of Athletes: Insights from Japanese Handball Players
Source: Microorganisms. 2024 Apr 11;12(4):781. doi: 10.3390/microorganisms12040781 (PMC11051819; doi:10.3390/microorganisms12040781)
Supplement: Supplementary file 1 [file microorganisms-12-00781-s001.zip › microorganisms-2952668-supplementary.pdf]

Supplementary Materials

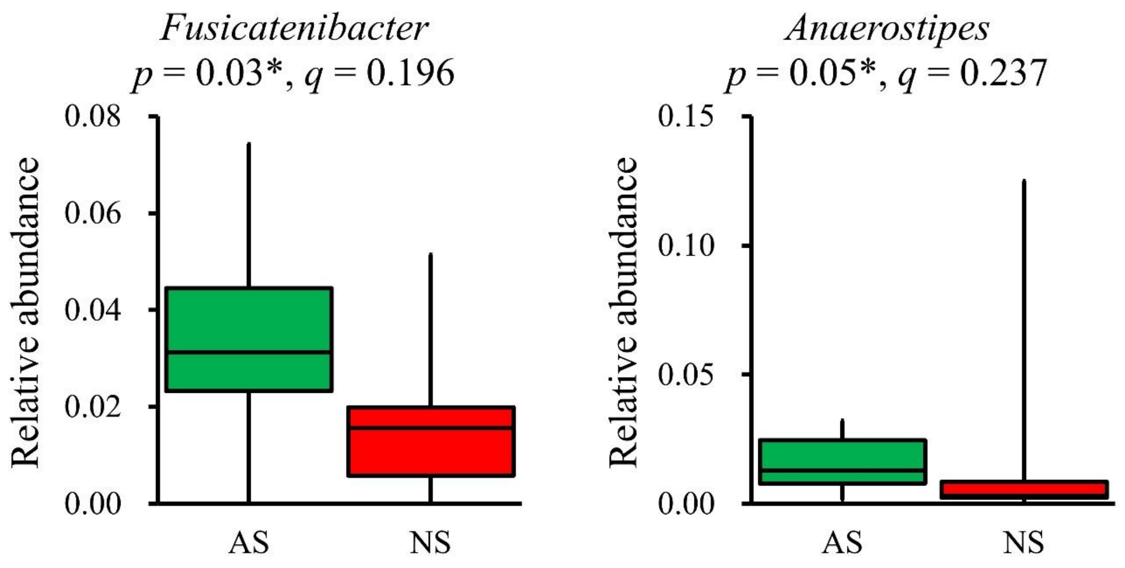

Supplemental Figure S1. *Fusicatenibacter* and *Anaerostipes* showed a tendency to differ between AS and NS in dataset 1

Among 19 dominant genera (>1%), *Fusicatenibacter* and *Anaerostipes* showed a tendency to differ between AS (n=17, green) and NS (n=10, red), as presented by box plots. Box plots show the median, as well as the lower and upper quartiles. Whiskers represent the minimum and maximum spread. Statistical analysis was performed using the Mann–Whitney U test and the Benjamini–Hochberg procedure. *Faecalibacterium* and *Streptococcus*, which were significantly different ($p<0.05$ and $q<0.05$), are presented in Figure 3. Abbreviations: Athletic subjects (AS); Non-athletic subjects (NS).

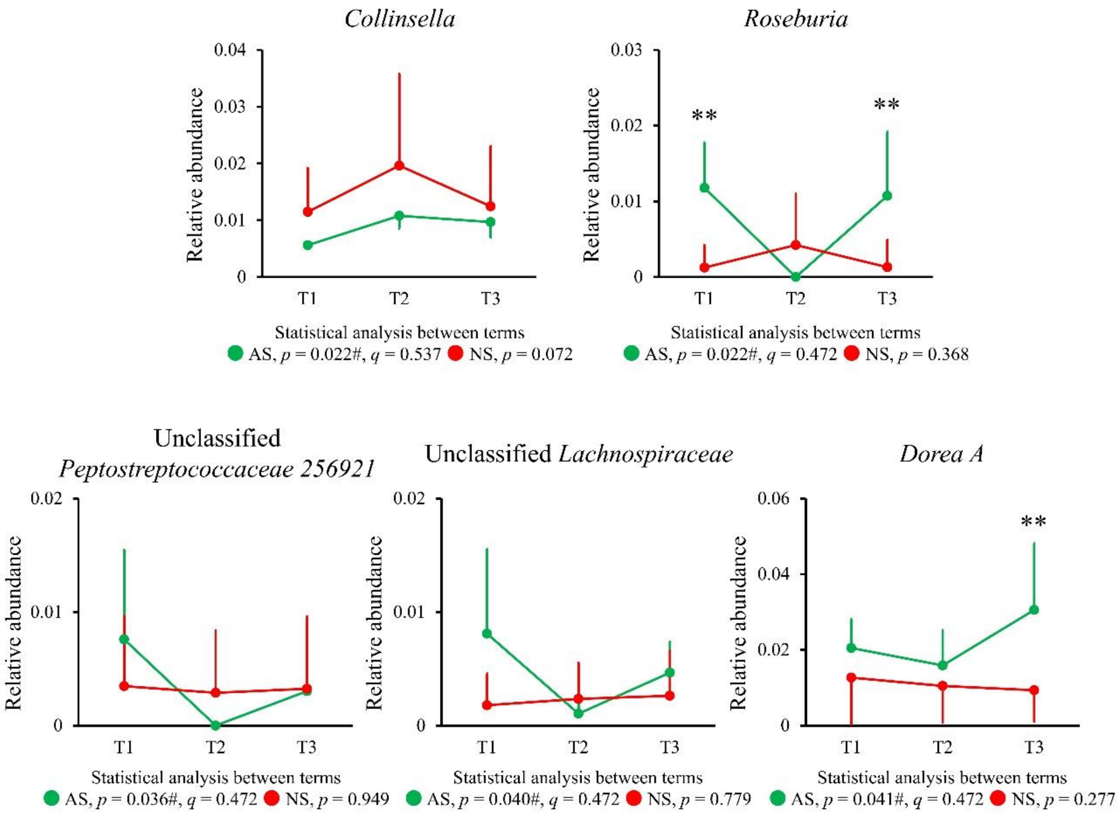

Supplemental Figure S2. Genera showed a tendency to differ between terms in dataset 2

We attempted to search for genera with significant changes between the athletic seasons and the off-season. Although none of the detected genera indicated significant longitudinal changes, *Collinsella*, *Roseburia*, unclassified *Peptostreptococcaceae 256921*, unclassified *Lachnospiraceae*, and *Dorea A* tended to change ($p<0.05$ and $q>0.05$). The p-values were assessed using the Mann–Whitney U test and the Friedman test for comparisons between groups (**$p<0.01$) and between terms (#$p<0.05$), respectively. The q-values were analyzed using the Benjamini–Hochberg procedure. The data are ex-pressed as the mean and standard deviation. Abbreviations: AS, Athletic subjects; NS, Non-athletic subjects; T1, term 1 (athletic season); T2, term 2 (off-season); T3, term 3 (athletic season).

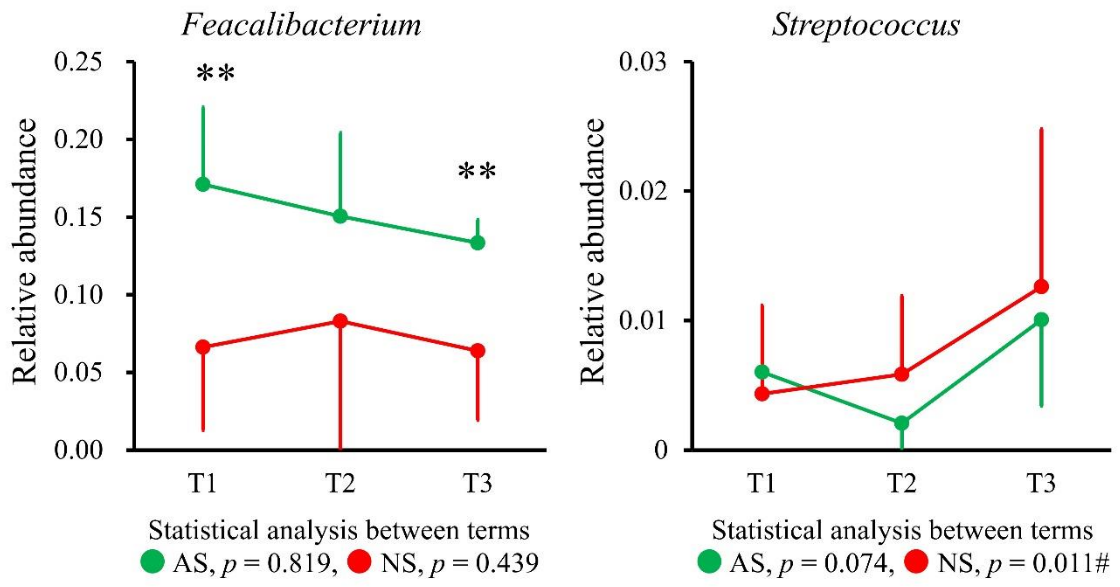

Supplemental Figure S3. Longitudinal analysis of the relative abundance of *Faecalibacterium* and *Streptococcus* between terms in dataset 2

Faecalibacterium and *Streptococcus*, which showed a significant difference in abundance in dataset 1 (Figure 3), are presented by the mean and standard deviation. The *p*-values were assessed using the Mann–Whitney U test and the Friedman test for comparisons between groups (**$p<0.01$) and between terms (#$p<0.05$), respectively. Abbreviations: AS, Athletic subjects; NS, Non-athletic subjects; T1, term 1 (athletic season); T2, term 2 (off-season); T3, term 3 (athletic season).

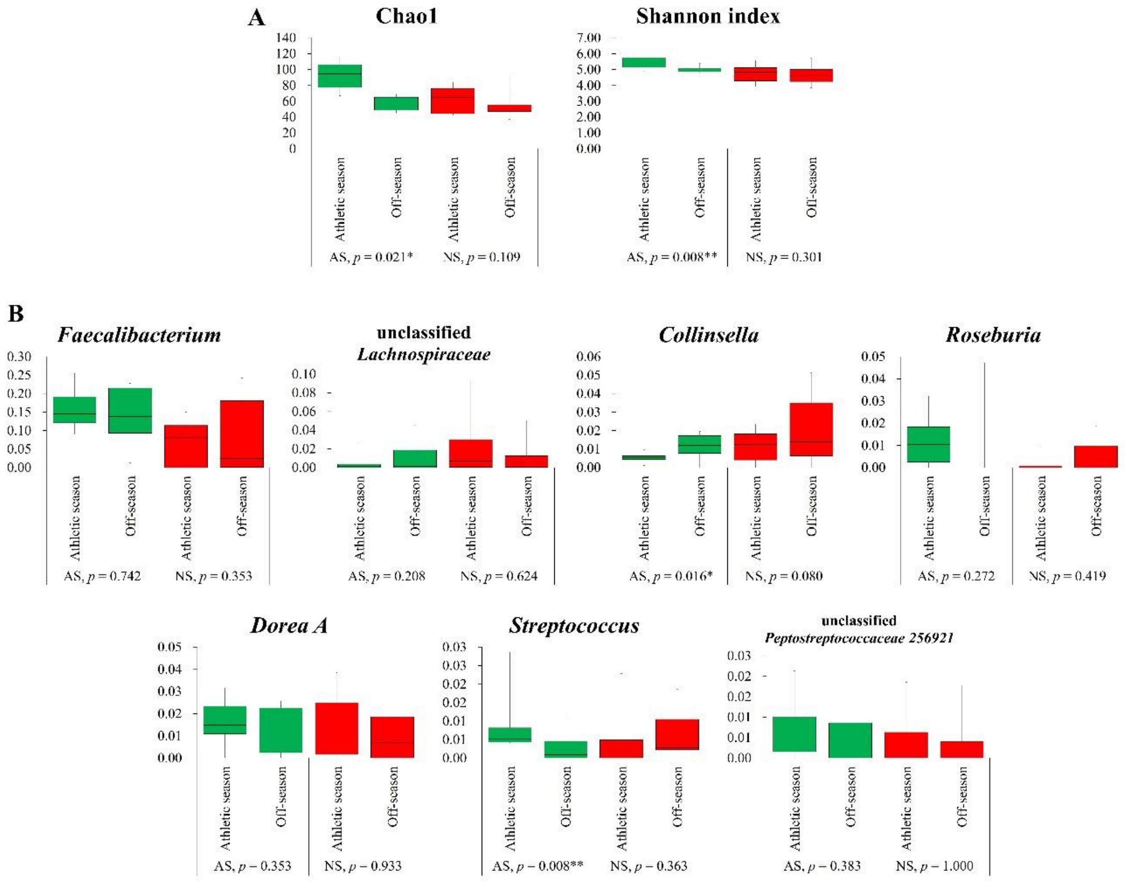

Supplemental Figure S4. Alpha-diversity and the noted genus compared between the athletic season and the off-season.

To revalidate the results obtained from the analysis using Dataset2 by increasing the sample size, we conducted an analysis using samples collected during two periods: T1 (the athletic season) and T2 (the offseason). The samples used for the analysis consisted of AS; Nimo5002, Nimo5004, Nimo5005, Nimo5011, Nimo5012, Nimo5016, Nimo5018, Nimo5022 (n=8) and NS; Nimo5009, Nimo5017, Nimo5023, Nimo5027, Nimo5029, Nimo5031, Nimo5032, Nimo5034, Nimo5035 (n=9). The results regarding (A) alpha-diversity and (B) bacterial genera, as also analyzed in Figure 4, Supplemental Figure S2 and S3, are presented using box plots. The box plots display the median, lower and upper quartiles, while the whiskers represent the minimum and maximum spread. Statistical analysis was performed using the paired t-test (*p<0.05, **p<0.01). Abbreviations: AS, Athletic subjects; NS, Non-athletic subjects; T1, term 1 (athletic season); T2, term 2 (off-season); T3, term 3 (athletic season).

Supplemental Table S1. Detailed background information for subjects

			Time of stool sampling					Background information						
ID	Group		Dataset 1	Dataset 2	Term 1 (T1)	Term 2 (T2)	Term 3 (T3)	Age	Sex	Height (cm)	Body weight (kg)	BMI (kg/m²)	Active time per day (e.g. sports and/or physical labor)	Sedentary time per day
Nimo5001	AS	Handball player	○		Feb-22			Twenties	Male	182	98	29.6	30 min - 3 hours	3-8 hours
Nimo5002	AS	Handball player	○	○	Nov-21	Apr-22	Oct-22	Thirties	Male	186	86	24.9	3 hours or more	3-8 hours
Nimo5003	AS	Handball player	○		Nov-21			Twenties	Male	170	74	25.6	30 min - 3 hours	3-8 hours
Nimo5004	AS	Handball player	○	○	Jan-22	Jun-22	Jul-22	Twenties	Male	190	100	27.7	3 hours or more	3-8 hours
Nimo5005	AS	Handball player	○	○	Feb-22	Apr-22	Aug-22	Twenties	Male	190	90	24.9	30 min - 3 hours	3-8 hours
Nimo5006	AS	Handball player	○		Nov-21			Twenties	Male	170	70	24.2	30 min - 3 hours	3-8 hours
Nimo5007	AS	Handball player	○		Nov-21			Twenties	Male	186	92	26.6	3 hours or more	3-8 hours
Nimo5008	AS	Handball player	○		Feb-22			Thirties	Male	183	96	28.7	3 hours or more	3-8 hours
Nimo5010	AS	Handball player	○		Nov-21			Twenties	Male	193	93	25.0	30 min - 3 hours	3-8 hours
Nimo5011	AS	Handball player	○	○	Nov-21	May-22	Oct-22	Thirties	Male	177	86	27.6	3 hours or more	3-8 hours
Nimo5012	AS	Handball player	○		Nov-21			Thirties	Male	185	87	25.4	30 min - 3 hours	3-8 hours
Nimo5013	AS	Handball player	○		Feb-22			Twenties	Male	179	82	25.6	30 min - 3 hours	3-8 hours
Nimo5016	AS	Handball player	○	○	Mar-22	May-22	Jul-22	Twenties	Male	183	89	26.3	30 min - 3 hours	3-8 hours
Nimo5018	AS	Handball player	○		Nov-21			Twenties	Male	178	81	25.6	30 min - 3 hours	Less than 3 hours
Nimo5019	AS	Handball player	○		Nov-21			Twenties	Male	185	88	25.7	30 min - 3 hours	3-8 hours
Nimo5020	AS	Handball player	○		Feb-22			Twenties	Male	192	99	26.9	30 min - 3 hours	Less than 3 hours
Nimo5022	AS	Handball player	○		Nov-21			Twenties	Male	175	78	25.3	30 min - 3 hours	3-8 hours
Nimo5009	NS	Team's staff	○	○	Mar-22	May-22	Sep-22	Thirties	Male	167	69	24.7	Less than 30 min	3-8 hours
Nimo5015	NS	Team's staff	○		Nov-21			Thirties	Male	186	88	25.4	30 min - 3 hours	3-8 hours
Nimo5017	NS	Subject not related to the team	○	○	Dec-21	Jun-22	Jul-22	Thirties	Male	189	88	24.6	Less than 30 min	Less than 3 hours
Nimo5023	NS	Subject not related to the team	○	○	Feb-22	May-22	Nov-22	Twenties	Male	175	62	20.2	30 min - 3 hours	3-8 hours
Nimo5027	NS	Subject not related to the team	○	○	Mar-22	May-22	Aug-22	Twenties	Male	168	74	26.2	30 min - 3 hours	3-8 hours
Nimo5029	NS	Subject not related to the team	○	○	Jan-22	May-22	Sep-22	Twenties	Male	175	65	21.2	Less than 30 min	8 hours or more
Nimo5031	NS	Subject not related to the team	○	○	Mar-22	May-22	Sep-22	Twenties	Male	173	59	19.7	30 min - 3 hours	3-8 hours
Nimo5032	NS	Subject not related to the team	○	○	Feb-22	Apr-22	Sep-22	Thirties	Male	172	64	21.5	Less than 30 min	8 hours or more
Nimo5034	NS	Subject not related to the team	○	○	Jan-22	Jun-22	Nov-22	Thirties	Male	189	75	21.0	Less than 30 min	8 hours or more
Nimo5035	NS	Subject not related to the team	○	○	Feb-22	Apr-22	Oct-22	Thirties	Male	172	62	21.0	Less than 30 min	8 hours or more

*Abbreviations: Athletic subjects (AS); Non-athletic subjects (NS); term 1 (T1); term 2 (T2); term 3 (T3).

Supplemental Table S2. Individual data regarding alpha-diversity

ID	Group	Time of stool sampling	Dataset 1	Dataset 2	faith pd	shannon entropy	chao1	pielou evenness	observed features
Nimo5001	AS	T1	○		15.6	5.23	83	0.83	78
Nimo5002	AS	T1	○	○	19.6	5.68	96	0.87	92
Nimo5002	AS	T2		○	13.4	5.11	50	0.91	50
Nimo5002	AS	T3		○	17.3	5.32	86	0.84	81
Nimo5003	AS	T1	○		16.8	5.15	91	0.80	84
Nimo5004	AS	T1	○	○	16.4	5.10	67	0.84	66
Nimo5004	AS	T2		○	15.1	5.01	68	0.83	67
Nimo5004	AS	T3		○	17.9	5.57	102	0.85	94
Nimo5005	AS	T1	○	○	18.9	5.36	93	0.83	90
Nimo5005	AS	T2		○	15.8	4.95	54	0.86	53
Nimo5005	AS	T3		○	18.8	5.44	91	0.84	90
Nimo5006	AS	T1	○		14.3	4.82	64	0.81	61
Nimo5007	AS	T1	○		15.7	5.18	87	0.82	82
Nimo5008	AS	T1	○		18.3	5.39	100	0.82	96
Nimo5010	AS	T1	○		14.3	5.11	77	0.82	74
Nimo5011	AS	T1	○	○	20.3	6.02	115	0.88	113
Nimo5011	AS	T2		○	16.5	5.09	69	0.84	68
Nimo5011	AS	T3		○	19.7	5.82	110	0.86	106
Nimo5012	AS	T1	○		16.4	5.45	97	0.84	90
Nimo5013	AS	T1	○		13.8	5.20	68	0.85	68
Nimo5016	AS	T1	○	○	16.3	5.23	85	0.83	80
Nimo5016	AS	T2		○	14.4	4.82	55	0.84	54
Nimo5016	AS	T3		○	17.5	5.55	90	0.86	88
Nimo5018	AS	T1	○		15.1	4.75	75	0.77	71
Nimo5019	AS	T1	○		13.3	4.79	57	0.83	56
Nimo5020	AS	T1	○		15.9	5.08	79	0.81	76
Nimo5022	AS	T1	○		19.5	5.78	109	0.86	103
Nimo5009	NS	T1	○	○	14.5	3.81	45	0.69	45
Nimo5009	NS	T2		○	12.5	3.53	37	0.69	35
Nimo5009	NS	T3		○	14.5	4.00	52	0.70	51
Nimo5015	NS	T1	○		21.3	5.76	102	0.87	99
Nimo5017	NS	T1	○	○	18.6	5.46	84	0.86	81
Nimo5017	NS	T2		○	16.6	5.39	94	0.84	87
Nimo5017	NS	T3		○	14.6	5.14	67	0.85	66
Nimo5023	NS	T1	○	○	12.9	4.30	43	0.79	43
Nimo5023	NS	T2		○	13.7	4.43	48	0.79	48
Nimo5023	NS	T3		○	13.2	4.59	50	0.82	49
Nimo5027	NS	T1	○	○	11.8	4.28	42	0.80	41
Nimo5027	NS	T2		○	14.1	4.61	54	0.80	53
Nimo5027	NS	T3		○	12.9	4.48	50	0.80	49
Nimo5029	NS	T1	○	○	18.0	5.04	76	0.82	70
Nimo5029	NS	T2		○	15.5	4.96	57	0.85	57
Nimo5029	NS	T3		○	19.3	5.20	91	0.81	86
Nimo5031	NS	T1	○	○	14.3	4.65	58	0.80	57
Nimo5031	NS	T2		○	12.7	4.43	46	0.80	46
Nimo5031	NS	T3		○	14.8	4.64	66	0.77	66
Nimo5032	NS	T1	○	○	16.9	5.17	76	0.83	75
Nimo5032	NS	T2		○	14.8	5.04	53	0.88	53
Nimo5032	NS	T3		○	15.5	4.31	63	0.73	59
Nimo5034	NS	T1	○	○	15.4	4.83	65	0.80	64
Nimo5034	NS	T2		○	12.3	4.05	48	0.73	46
Nimo5034	NS	T3		○	12.2	4.23	42	0.80	39
Nimo5035	NS	T1	○	○	15.5	4.93	72	0.81	69
Nimo5035	NS	T2		○	14.2	4.82	52	0.85	52
Nimo5035	NS	T3		○	15.1	4.88	74	0.79	71

*Abbreviations: Athletic subjects (AS); Non-athletic subjects (NS); term 1 (T1); term 2 (T2); term 3 (T3).

Supplemental Table S3. Individual data regarding relative abundance at the genus level

ID	Nimo5001	Nimo5002			Nimo5003	Nimo5004			[illegible]
Time of stool sampling	T1	T1	T2	T3	T1	T1	T2	T3	[illegible]
Group	AS	AS	AS	AS	AS	AS	AS	AS	[illegible]
Dataset 1	○	○			○	○			[illegible]
Dataset 2		○	○	○		○	○	○	[illegible]
d__Bacteria|__|__|__|__|__	0.000	0.001	0.000	0.000	0.001	0.000	0.000	0.000	[illegible]
d__Bacteria|p__|c__|o__|f__|g__	0.000	0.001	0.000	0.000	0.000	0.000	0.000	0.000	[illegible]
d__Bacteria|p__Actinobacteriota|c__Actinomycetia|o__Actinomycetales|f__Bifidobacteriaceae|g__Bifidobacterium_388775	0.005	0.019	0.008	0.011	0.182	0.017	0.047	0.054	[illegible]
d__Bacteria|p__Actinobacteriota|c__Actinomycetia|o__Mycobacteriales|f__Mycobacteriaceae|g__Corynebacterium	0.000	0.000	0.000	0.000	0.000	0.000	0.000	0.000	[illegible]
d__Bacteria|p__Actinobacteriota|c__Coriobacteriia|o__Coriobacteriales|__|__	0.000	0.000	0.000	0.000	0.000	0.000	0.000	0.000	[illegible]
d__Bacteria|p__Actinobacteriota|c__Coriobacteriia|o__Coriobacteriales|f__Atopobiaceae|g__	0.000	0.000	0.000	0.000	0.000	0.000	0.000	0.000	[illegible]
d__Bacteria|p__Actinobacteriota|c__Coriobacteriia|o__Coriobacteriales|f__Atopobiaceae|g__Parolsenella	0.000	0.000	0.000	0.000	0.000	0.000	0.000	0.000	[illegible]
d__Bacteria|p__Actinobacteriota|c__Coriobacteriia|o__Coriobacteriales|f__Atopobiaceae|g__Thermophilibacter	0.000	0.000	0.000	0.000	0.001	0.000	0.000	0.000	[illegible]
d__Bacteria|p__Actinobacteriota|c__Coriobacteriia|o__Coriobacteriales|f__Coriobacteriaceae|g__Collinsella	0.019	0.006	0.009	0.007	0.012	0.006	0.012	0.012	[illegible]
d__Bacteria|p__Actinobacteriota|c__Coriobacteriia|o__Coriobacteriales|f__Eggerthellaceae|__	0.000	0.000	0.000	0.000	0.000	0.000	0.000	0.000	[illegible]
d__Bacteria|p__Actinobacteriota|c__Coriobacteriia|o__Coriobacteriales|f__Eggerthellaceae|g__Adlercreutzia_404257	0.000	0.000	0.000	0.000	0.000	0.000	0.000	0.000	[illegible]
d__Bacteria|p__Actinobacteriota|c__Coriobacteriia|o__Coriobacteriales|f__Eggerthellaceae|g__CAG-1427	0.000	0.001	0.000	0.000	0.001	0.000	0.000	0.000	[illegible]
d__Bacteria|p__Actinobacteriota|c__Coriobacteriia|o__Coriobacteriales|f__Eggerthellaceae|g__Eggerthella	0.002	0.000	0.000	0.000	0.000	0.000	0.000	0.000	[illegible]
d__Bacteria|p__Actinobacteriota|c__Coriobacteriia|o__Coriobacteriales|f__Eggerthellaceae|g__Ellagibacter	0.000	0.000	0.000	0.000	0.000	0.000	0.000	0.001	[illegible]
d__Bacteria|p__Actinobacteriota|c__Coriobacteriia|o__Coriobacteriales|f__Eggerthellaceae|g__Senegalimassilia	0.000	0.000	0.000	0.001	0.000	0.000	0.000	0.000	[illegible]
d__Bacteria|p__Actinobacteriota|c__Coriobacteriia|o__Coriobacteriales|f__Eggerthellaceae|g__Slackia_A	0.000	0.000	0.000	0.000	0.000	0.000	0.000	0.000	[illegible]
d__Bacteria|p__Bacteroidota|c__Bacteroidia|__|__|__	0.000	0.000	0.000	0.000	0.000	0.000	0.000	0.000	[illegible]
d__Bacteria|p__Bacteroidota|c__Bacteroidia|o__Bacteroidales|__|__	0.000	0.000	0.000	0.000	0.000	0.000	0.000	0.000	[illegible]
d__Bacteria|p__Bacteroidota|c__Bacteroidia|o__Bacteroidales|f__Bacteroidaceae|__	0.000	0.000	0.001	0.000	0.000	0.000	0.000	0.000	[illegible]
d__Bacteria|p__Bacteroidota|c__Bacteroidia|o__Bacteroidales|f__Bacteroidaceae|g__Alloprevotella	0.000	0.111	0.080	0.139	0.000	0.000	0.000	0.000	[illegible]
d__Bacteria|p__Bacteroidota|c__Bacteroidia|o__Bacteroidales|f__Bacteroidaceae|g__Bacteroides_H	0.114	0.032	0.041	0.005	0.092	0.030	0.093	0.052	[illegible]
d__Bacteria|p__Bacteroidota|c__Bacteroidia|o__Bacteroidales|f__Bacteroidaceae|g__Paraprevotella	0.000	0.005	0.017	0.012	0.000	0.019	0.007	0.022	[illegible]
d__Bacteria|p__Bacteroidota|c__Bacteroidia|o__Bacteroidales|f__Bacteroidaceae|g__Phocaeicola_A_858004	0.089	0.069	0.065	0.057	0.089	0.146	0.182	0.157	[illegible]
d__Bacteria|p__Bacteroidota|c__Bacteroidia|o__Bacteroidales|f__Bacteroidaceae|g__Prevotella	0.000	0.066	0.053	0.090	0.000	0.019	0.003	0.004	[illegible]
d__Bacteria|p__Bacteroidota|c__Bacteroidia|o__Bacteroidales|f__Barnesiellaceae|g__Barnesiella	0.000	0.000	0.000	0.000	0.003	0.001	0.000	0.001	[illegible]
d__Bacteria|p__Bacteroidota|c__Bacteroidia|o__Bacteroidales|f__Coprobacteraceae|g__Coprobacter	0.000	0.000	0.000	0.000	0.000	0.000	0.000	0.000	[illegible]
d__Bacteria|p__Bacteroidota|c__Bacteroidia|o__Bacteroidales|f__Marinifilaceae|g__Butyricimonas	0.002	0.000	0.000	0.000	0.000	0.000	0.000	0.001	[illegible]
d__Bacteria|p__Bacteroidota|c__Bacteroidia|o__Bacteroidales|f__Marinifilaceae|g__Odoribacter_865974	0.000	0.001	0.000	0.000	0.001	0.000	0.000	0.000	[illegible]
[illegible]	[illegible]	[illegible]	[illegible]	[illegible]	[illegible]	[illegible]	[illegible]	[illegible]	[illegible]
d__Bacteria|p__Firmicutes_D|c__Bacilli|o__Lactobacillales|f__Lactobacillaceae|g__Limosilactobacillus	0.000	0.000	0.000	0.000	[illegible]	[illegible]	[illegible]	[illegible]	[illegible]
d__Bacteria|p__Firmicutes_D|c__Bacilli|o__Lactobacillales|f__Streptococcaceae|g__Lactococcus_A_346120	0.001	0.000	0.000	0.000	[illegible]	[illegible]	[illegible]	[illegible]	[illegible]
d__Bacteria|p__Firmicutes_D|c__Bacilli|o__Lactobacillales|f__Streptococcaceae|g__Streptococcus	0.010	0.007	0.004	0.010	[illegible]	[illegible]	[illegible]	[illegible]	[illegible]
d__Bacteria|p__Firmicutes_D|c__Bacilli|o__ML615J-28|f__CAG-313|g__CAG-313	0.000	0.000	0.000	0.000	[illegible]	[illegible]	[illegible]	[illegible]	[illegible]
d__Bacteria|p__Firmicutes_D|c__Bacilli|o__RF39|f__UBA660|g__CAG-302	0.000	0.000	0.000	0.000	[illegible]	[illegible]	[illegible]	[illegible]	[illegible]
d__Bacteria|p__Firmicutes_D|c__Bacilli|o__Staphylococcales|f__Gemellaceae|g__Gemella	0.000	0.000	0.000	0.000	[illegible]	[illegible]	[illegible]	[illegible]	[illegible]
d__Bacteria|p__Fusobacteriota|c__Fusobacteriia|o__Fusobacteriales_993521|f__Fusobacteriaceae_993521|g__Fusobacterium_A	0.090	0.000	0.000	0.000	[illegible]	[illegible]	[illegible]	[illegible]	[illegible]
d__Bacteria|p__Proteobacteria|c__Alphaproteobacteria|o__RF32|f__CAG-239|g__51-20	0.000	0.000	0.000	0.000	[illegible]	[illegible]	[illegible]	[illegible]	[illegible]
d__Bacteria|p__Proteobacteria|c__Alphaproteobacteria|o__RF32|f__CAG-239|g__CAG-267	0.000	0.000	0.000	0.000	[illegible]	[illegible]	[illegible]	[illegible]	[illegible]
d__Bacteria|p__Proteobacteria|c__Alphaproteobacteria|o__RF32|f__CAG-239|g__Scatocola	0.000	0.000	0.000	0.000	[illegible]	[illegible]	[illegible]	[illegible]	[illegible]
d__Bacteria|p__Proteobacteria|c__Gammaproteobacteria|__|__|__	0.000	0.000	0.000	0.000	[illegible]	[illegible]	[illegible]	[illegible]	[illegible]
d__Bacteria|p__Proteobacteria|c__Gammaproteobacteria|o__Burkholderiales_595427|f__Burkholderiaceae_A_595427|g__Aphodousia	0.000	0.000	0.000	0.000	[illegible]	[illegible]	[illegible]	[illegible]	[illegible]
d__Bacteria|p__Proteobacteria|c__Gammaproteobacteria|o__Burkholderiales_595427|f__Burkholderiaceae_A_595427|g__Duodenibacillus	0.000	0.004	0.006	0.000	[illegible]	[illegible]	[illegible]	[illegible]	[illegible]
d__Bacteria|p__Proteobacteria|c__Gammaproteobacteria|o__Burkholderiales_595427|f__Burkholderiaceae_A_595427|g__Mesosutterella	0.000	0.000	0.000	0.000	[illegible]	[illegible]	[illegible]	[illegible]	[illegible]
d__Bacteria|p__Proteobacteria|c__Gammaproteobacteria|o__Burkholderiales_595427|f__Burkholderiaceae_A_595427|g__Parasutterella	0.000	0.001	0.000	0.004	[illegible]	[illegible]	[illegible]	[illegible]	[illegible]
d__Bacteria|p__Proteobacteria|c__Gammaproteobacteria|o__Burkholderiales_595427|f__Burkholderiaceae_A_595427|g__Sutterella	0.017	0.000	0.000	0.000	[illegible]	[illegible]	[illegible]	[illegible]	[illegible]
d__Bacteria|p__Proteobacteria|c__Gammaproteobacteria|o__Enterobacterales_A_737866|f__Enterobacteriaceae_A|__	0.000	0.000	0.000	0.003	[illegible]	[illegible]	[illegible]	[illegible]	[illegible]
d__Bacteria|p__Proteobacteria|c__Gammaproteobacteria|o__Enterobacterales_A_737866|f__Enterobacteriaceae_A|g__Citrobacter_A_692098	0.000	0.000	0.000	0.000	[illegible]	[illegible]	[illegible]	[illegible]	[illegible]
d__Bacteria|p__Proteobacteria|c__Gammaproteobacteria|o__Enterobacterales_A_737866|f__Pasteurellaceae|g__Haemophilus_D_735815	0.000	0.000	0.000	0.002	[illegible]	[illegible]	[illegible]	[illegible]	[illegible]
d__Bacteria|p__Proteobacteria|c__Gammaproteobacteria|o__Enterobacterales_A_737866|f__Succinivibrionaceae|g__Succinatimonas	0.000	0.000	0.000	0.000	[illegible]	[illegible]	[illegible]	[illegible]	[illegible]
d__Bacteria|p__Verrucomicrobiota|c__Verrucomicrobiae|o__Verrucomicrobiales|f__Akkermansiaceae|g__Akkermansia	0.001	0.000	0.000	0.000	[illegible]	[illegible]	[illegible]	[illegible]	[illegible]

*Abbreviations: Athletic subjects (AS); Non-athletic subjects (NS); term 1 (T1); term 2 (T2); term 3 (T3).

Supplemental Table S4. The relative abundance at the genus level (>1%) for dataset 1

	Relative abundance				Detection ratio			
	AS Mean ± SD	NS Mean ± SD	p-values	q-values	AS	NS	p-values	q-values
Phocaeicola A 858004	0.131 ± 0.054	0.134 ± 0.091	0.863	0.980	100.0%	90.0%	0.370	0.986
Faecalibacterium	0.145 ± 0.054	0.070 ± 0.052	0.003 **	0.024 ‡	94.1%	70.0%	0.128	0.812
Blautia A 141781	0.079 ± 0.021	0.083 ± 0.029	0.941	0.980	100.0%	100.0%	1.000	1.000
Bacteroides H	0.061 ± 0.044	0.116 ± 0.100	0.223	0.471	100.0%	100.0%	1.000	1.000
Bifidobacterium 388775	0.057 ± 0.055	0.096 ± 0.071	0.141	0.383	100.0%	90.0%	0.370	0.986
Prevotella	0.053 ± 0.104	0.031 ± 0.057	0.933	0.980	41.2%	40.0%	1.000	1.000
Gemmiger A 73129	0.028 ± 0.021	0.040 ± 0.047	0.633	0.802	94.1%	80.0%	0.535	1.000
Ruminococcus E	0.015 ± 0.019	0.060 ± 0.064	0.164	0.390	52.9%	60.0%	1.000	1.000
Agathobacter 164117	0.042 ± 0.041	0.017 ± 0.023	0.113	0.358	70.6%	40.0%	0.224	0.986
Fusicatenibacter	0.032 ± 0.019	0.017 ± 0.014	0.033 *	0.207	88.2%	90.0%	1.000	1.000
Anaerostipes	0.016 ± 0.009	0.018 ± 0.036	0.047 *	0.225	100.0%	80.0%	0.128	0.812
Ruminococcus B	0.010 ± 0.013	0.022 ± 0.032	0.410	0.708	58.8%	70.0%	0.692	1.000
Faecalibacillus	0.015 ± 0.014	0.017 ± 0.017	0.980	0.980	76.5%	60.0%	0.415	0.986
Parabacteroides B 862066	0.012 ± 0.008	0.016 ± 0.010	0.366	0.695	94.1%	90.0%	1.000	1.000
Fusobacterium A	0.016 ± 0.041	0.003 ± 0.005	0.580	0.787	29.4%	20.0%	0.678	1.000
Streptococcus	0.018 ± 0.020	0.004 ± 0.007	0.001 **	0.024 ‡	100.0%	60.0%	0.012 *	0.228
Alloprevotella	0.022 ± 0.048	0.000 ± 0.000	0.113	0.358	23.5%	0.0%	0.264	0.986
Dorea A	0.014 ± 0.010	0.012 ± 0.012	0.530	0.775	88.2%	80.0%	0.613	1.000
Unclassified *Lachnospiraceae*	0.008 ± 0.013	0.018 ± 0.027	0.494	0.775	76.5%	70.0%	1.000	1.000

The relative abundance at the genus level (>1%) for dataset 1.

*Abbreviations: Athletic subjects (AS), Non-athletic subjects (NS), standard deviation (SD).

The p-values and q-values were statistically analyzed using the Mann–Whitney U test and Benjamini–Hochberg procedure, respectively.

*p <0.05, **p <0.001, ‡q <0.05

Supplemental table S5. DDBJ accession numbers corresponding to 16S rRNA gene sequence data

ID	Group	Time of stool sampling	Dataset 1	Dataset 2	BioProject ID	BioSample ID
Nimo5001	AS	T1	○		PRJDB17286	SAMD00729907
Nimo5002	AS	T1	○	○	PRJDB17286	SAMD00729908
Nimo5002	AS	T2		○	PRJDB17286	SAMD00729909
Nimo5002	AS	T3		○	PRJDB17286	SAMD00729910
Nimo5003	AS	T1	○		PRJDB17286	SAMD00729911
Nimo5004	AS	T1	○	○	PRJDB17286	SAMD00729912
Nimo5004	AS	T2		○	PRJDB17286	SAMD00729913
Nimo5004	AS	T3		○	PRJDB17286	SAMD00729914
Nimo5005	AS	T1	○	○	PRJDB17286	SAMD00729915
Nimo5005	AS	T2		○	PRJDB17286	SAMD00729916
Nimo5005	AS	T3		○	PRJDB17286	SAMD00729917
Nimo5006	AS	T1	○		PRJDB17286	SAMD00729918
Nimo5007	AS	T1	○		PRJDB17286	SAMD00729919
Nimo5008	AS	T1	○		PRJDB17286	SAMD00729920
Nimo5010	AS	T1	○		PRJDB17286	SAMD00729924
Nimo5011	AS	T1	○	○	PRJDB17286	SAMD00729925
Nimo5011	AS	T2		○	PRJDB17286	SAMD00729926
Nimo5011	AS	T3		○	PRJDB17286	SAMD00729927
Nimo5012	AS	T1	○		PRJDB17286	SAMD00729928
Nimo5013	AS	T1	○		PRJDB17286	SAMD00729929
Nimo5016	AS	T1	○	○	PRJDB17286	SAMD00729931
Nimo5016	AS	T2		○	PRJDB17286	SAMD00729932
Nimo5016	AS	T3		○	PRJDB17286	SAMD00729933
Nimo5018	AS	T1	○		PRJDB17286	SAMD00729937
Nimo5019	AS	T1	○		PRJDB17286	SAMD00729938
Nimo5020	AS	T1	○		PRJDB17286	SAMD00729939
Nimo5022	AS	T1	○		PRJDB17286	SAMD00729940
Nimo5009	NS	T1	○	○	PRJDB17286	SAMD00729921
Nimo5009	NS	T2		○	PRJDB17286	SAMD00729922
Nimo5009	NS	T3		○	PRJDB17286	SAMD00729923
Nimo5015	NS	T1	○		PRJDB17286	SAMD00729930
Nimo5017	NS	T1	○	○	PRJDB17286	SAMD00729934
Nimo5017	NS	T2		○	PRJDB17286	SAMD00729935
Nimo5017	NS	T3		○	PRJDB17286	SAMD00729936
Nimo5023	NS	T1	○	○	PRJDB17286	SAMD00729941
Nimo5023	NS	T2		○	PRJDB17286	SAMD00729942
Nimo5023	NS	T3		○	PRJDB17286	SAMD00729943
Nimo5027	NS	T1	○	○	PRJDB17286	SAMD00729944
Nimo5027	NS	T2		○	PRJDB17286	SAMD00729945
Nimo5027	NS	T3		○	PRJDB17286	SAMD00729946
Nimo5029	NS	T1	○	○	PRJDB17286	SAMD00729947
Nimo5029	NS	T2		○	PRJDB17286	SAMD00729948
Nimo5029	NS	T3		○	PRJDB17286	SAMD00729949
Nimo5031	NS	T1	○	○	PRJDB17286	SAMD00729950
Nimo5031	NS	T2		○	PRJDB17286	SAMD00729951
Nimo5031	NS	T3		○	PRJDB17286	SAMD00729952
Nimo5032	NS	T1	○	○	PRJDB17286	SAMD00729953
Nimo5032	NS	T2		○	PRJDB17286	SAMD00729954
Nimo5032	NS	T3		○	PRJDB17286	SAMD00729955
Nimo5034	NS	T1	○	○	PRJDB17286	SAMD00729956
Nimo5034	NS	T2		○	PRJDB17286	SAMD00729957
Nimo5034	NS	T3		○	PRJDB17286	SAMD00729958
Nimo5035	NS	T1	○	○	PRJDB17286	SAMD00729959
Nimo5035	NS	T2		○	PRJDB17286	SAMD00729960
Nimo5035	NS	T3		○	PRJDB17286	SAMD00729961

*Abbreviations: Athletic subjects (AS); Non-athletic subjects (NS); term 1 (T1); term 2 (T2); term 3 (T3).
